# Optimally conductive networks in randomly dispersed CNT:graphene hybrids

**DOI:** 10.1038/srep16568

**Published:** 2015-11-13

**Authors:** Wonbo Shim, Youbin Kwon, Seung-Yeol Jeon, Woong-Ryeol Yu

**Affiliations:** 1 Department of Materials Science and Engineering and Research Institute of Advanced Materials (RIAM), Seoul National University, Seoul, Korea

## Abstract

A predictive model is proposed that quantitatively describes the synergistic behavior of the electrical conductivities of CNTs and graphene in CNT:graphene hybrids. The number of CNT-to-CNT, graphene-to-graphene, and graphene-to-CNT contacts is calculated assuming a random distribution of CNTs and graphene particles in the hybrids and using an orientation density function. Calculations reveal that the total number of contacts reaches a maximum at a specific composition and depends on the particle sizes of the graphene and CNTs. The hybrids, prepared using inkjet printing, are distinguished by higher electrical conductivities than that of 100% CNT or graphene at certain composition ratios. These experimental results provide strong evidence that this approach involving constituent element contacts is suitable for investigating the properties of particulate hybrid materials.

Carbon nanotubes (CNTs) and graphene have been intensively studied for various applications^1^,^2^,^3^,^4^,^5^ including supercapacitors^6^,^7^,^8^, field-emission devices^9^,^10^, and sensor devices^11^ because of their excellent mechanical, thermal, electrical, and optical properties. Thin films are the most versatile form for such applications, so various processing methods have been developed to fabricate thin films from graphene and CNT solutions, including filtration^12^,^13^, dip coating, electrophoretic deposition^14^, and inkjet printing^15^,^16^,^17^. These solution-based processing methods are advantageous for large-scale and low-cost manufacturing, but have some serious drawbacks. For example, graphene films made from solutions have lower electrical conductivities than the theoretical value, presumably because of poor interlayer junction resistance^18^. To overcome this problem, CNT:graphene hybrids have been suggested because the CNTs could then form conductive paths between the graphene flakes^19^,^20^.

CNT:graphene hybrids have been studied in many researches due to its enhanced properties than that of graphene and CNT itself. In some work, it was found that the thermal conductivity increases by hybridizing CNT/graphene^21^. In that work, they claimed that the enhancement of thermal conductivity in CNT:graphene hybrid is due to bridging of graphene flakes by CNTs. Most of the works related to CNT:graphene hybrids is about its electrical conductivity. In case of CNT:graphene hybrids in a film form, the CNT:graphene hybrid films have higher electrical conductivities than pure graphene films^12^,^22^,^23^,^24^,^25^,^26^,^27^,^28^,^29^. Additionally, in some researches, it was found that the CNT:graphene films with graphene and CNTs mixed and dispersed in the same layer have much higher electrical conductivities than graphene films coated with CNTs^23^. The improvement in the electrical conductivity by hybridization is also found in the CNT:graphene hybrids in fiber form^30^,^31^. The proposed mechanism for this improvement is that the CNTs are acting as conducting bridges between the graphene flakes^27^,^30^,^32^,^33^,^34^. CNT:graphene hybrids also have higher electrical conductivities than pure CNT films^23^,^24^,^25^,^28^,^29^. King *et al.* suggested that the synergism of the conductivities is derived from the graphene particles filling the space between the CNTs and thereby providing a lower junction resistance between the graphene and the CNTs. These two explanations for the synergistically improved electrical conductivity of CNT:graphene hybrids, i.e., CNTs acting as conducting bridges between the graphene flakes and graphene particles filling the space between CNTs, have the common feature of an increased number of contacts between the graphene particles and the CNTs. This study aimed to establish theoretical and experimental explanations for the synergistic effect. CNT:graphene hybrids prepared by simple dispersion and a film processing method were used in the experimental work.

Various processing methods have been developed to make CNT:graphene hybrid films. Vacuum filtration was the first solution approach^24^,^32^. Spin coating and solution casting have been used more recently^12^,^20^,^35^. Sequential self-assembly of graphene and CNT on a substrate have also been reported^36^. The alternative solid-phase composition method, i.e., attaching a solid graphene film to a solid CNT film^19^, has been used in various applications such as transparent electrodes, field effect transistors, and supercapacitors^23^,^36^. Recently, inkjet printing has been employed as a film processing method because of its simplicity and ability to make complex patterns; this approach has been used in applications such as memory devices, solar cells, organic thin film transistors, and light emitting diodes^37^. Inkjet printing was used in this study to prepare CNT:graphene hybrids from separate solutions, i.e., inks. It is important to avoid agglomeration of the particles in their respective solutions. Ball-milling, ultrasonication, or surfactant treatment^15^,^17^,^38^,^39^ have been previously used to prepare agglomeration-free graphene and CNT inks. However, in this study, a simple wrapping technique was used to prepare stable CNT:graphene hybrid inks^40^.

This paper presents a predictive model for quantitatively explaining the synergistic behavior of CNT:graphene hybrids. A quantitative analysis carried out using the model demonstrated that the hybrids can exhibit higher electrical conductivities than pure CNT or graphene films at certain composition ratios. CNT:graphene hybrids prepared by inkjet printing were used to validate the predictive model.

## Results

### A predictive model for the electrical conductivity of CNT:graphene hybrids

A predictive model was developed to explain the synergistic behavior of graphene and CNT electrical conductivities in CNT:graphene hybrids. The conductivity of these hybrids is governed by inter-particle contacts. A random stick network model can be used to estimate the electrical conductivity of a CNT film^41^. This model assumes that a CNT behaves as a conductive stick and that an assembly of many conductive sticks (e.g., a film) is randomly distributed in two dimensions. The electrical conductivity of a CNT film can then be determined according to Equation (1):


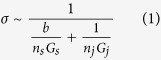


where *n*_*s*_ is the number of conductive sticks and *n*_*j*_ is the number of their junctions, i.e., the number of stick-to-stick contacts. Here, *b* is a constant, *G*_*s*_ is the stick conductance, and *G*_*j*_ is the junction conductance. Because the conductivities of individual CNTs and graphene are much higher than the conductivity of their junctions (contacts)^42^,^43^, Equation (1) can be simplified to 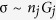 for CNT:graphene hybrids. On this basis, the electrical conductivity of CNT:graphene hybrids is proportional to the number of CNT-to-CNT, graphene-to-graphene, and graphene-to-CNT contacts. Furthermore, because the conductivity of a hybrid increases with increasing numbers of contacts made by the individual elements in the hybrid, the conductivity of the hybrid is maximized when the number of contacts is maximized. Consequently, the conductivity of CNT:graphene hybrids can be estimated by calculating all of the contacts made by the graphene and the CNTs.

**Figure 1:**
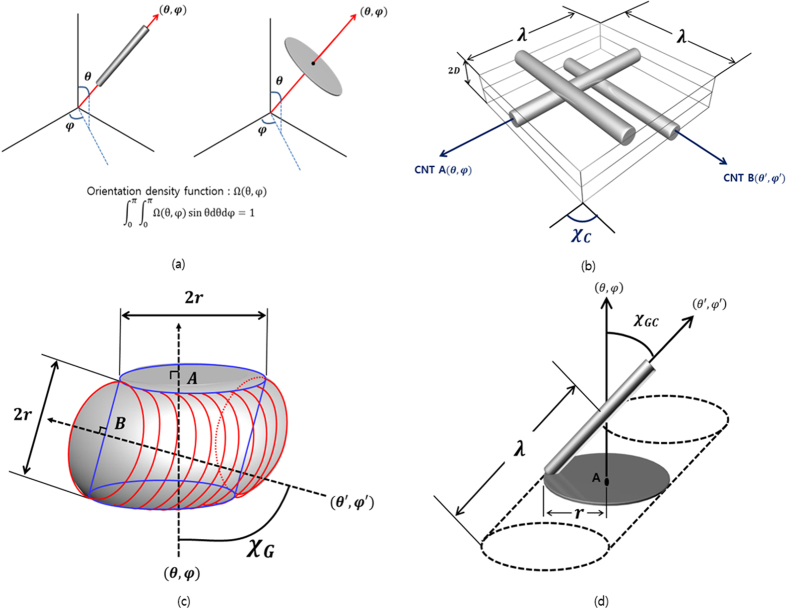
A model for calculating the number of CNT and graphene interparticle contacts. (**a**) Orientation density function describing the orientation of individual CNT and graphene particles in space. (**b**) The parallelepiped formed by a CNT *B* 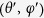 moving around a fixed CNT *A* 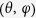 while maintaining contact^44^. (**c**) Contact of a graphene particle 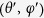 with graphene particle *A* 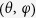, which is in a fixed position when the center of the mass of the graphene particle enters the region surrounded by the blue lines. (**d**) Contact of CNT 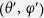 with the graphene particle 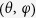 when the center of mass of the CNT enters into the region surrounded by the dotted lines.

Many studies have focused on predicting the mechanical properties of short-fiber assemblies. Calculation of the number of fiber-to-fiber contacts is very important because external loads are transferred between the short fibers through their contacts. Komori *et al.* defined the orientation of fibers in space using two angles (Fig. 1a) and developed a model to calculate the number of fiber-to-fiber contacts^44^. This model is used here to calculate the number of CNT-to-CNT contacts by considering the CNTs as short fibers. The Komori model is modified to accommodate different geometrical entities, i.e., CNT-to-graphene and graphene-to-graphene contacts. Details on these modifications are provided in the Supporting Information, and are described briefly here.

Assuming CNTs are straight cylinders of constant length and diameter, the contact condition can be stated as follows: CNT *B* will contact CNT *A* when the center of mass of the CNT *B* enters into the region shown in Fig. 1b. By calculating the volume of the region and the probability of the contact, the number of CNT-to-CNT contacts is given by:


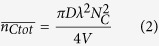


where *D* and *λ* are the diameter and length of the CNTs and *N*_*c*_ is the number of CNTs in the volume *V*. To estimate the number of graphene-to-graphene contacts, graphene is considered to be a disk with a radius of *r* and negligible thickness. Figure 1c is used to derive the contact condition, from which the number of graphene-to-graphene contacts is:


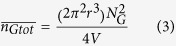


where *N*_*G*_ is the total number of graphene particles in the volume *V*. Next, consider the number of CNT-to-graphene contacts. The contact condition under which a CNT will contact a graphene particle can be similarly derived using Fig. 1d. The number of CNT-to-graphene contacts is then given by:


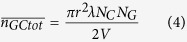


Summing Equations 2, 3, 4 provides the total number of contacts between the CNTs and graphene particles in a hybrid, i.e.:


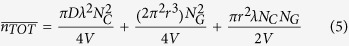


**Figure 2:**
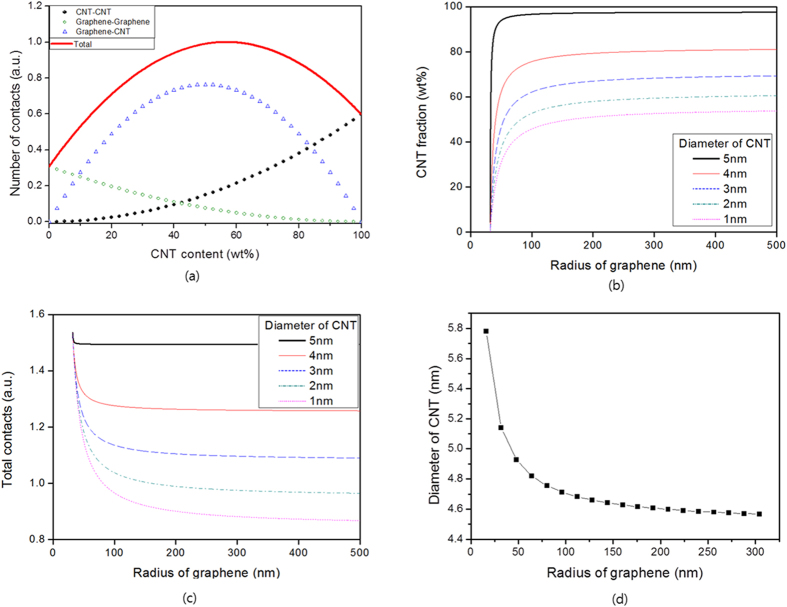
Calculated number of contacts made by CNT and graphene particles in their hybrid assembly. (**a**) The number of CNT-to-CNT, graphene-to-graphene, and graphene-to-CNT contacts in the CNT:graphene hybrids as a function of the CNT content. In this system, 56.7 wt% is the CNT fraction that can bring about the maximum number of contacts in the modeled CNT:graphene hybrids. At this concentration, the proportion of graphene-to-CNT contact is the largest (75.0%), followed by CNT-to-CNT (19.2%), and graphene-to-graphene (5.8%) contacts. A synergistic behavior of CNT:graphene hybrids can be observed at this CNT content. (**b**) The effect of graphene particle radius and CNT diameter on CNT content at the maximum number of contacts. (**c**) The number of contacts calculated for different graphene and CNT sizes in (**b**). (d) Relation between the diameter of CNT and radius of graphene when the maximum number of contacts occurs at CNT content of 89%.

Equation (5) assumes that the number of CNTs and graphene particles and their geometrical parameters are known in a specific volume. The morphologies and sizes of the CNTs and graphene are assigned as follows. Multi-walled CNTs (MWCNTs) having five walls and a length and diameter of 5 μm and 2 nm, respectively, are considered. The radius of a single-layered disk-shaped graphene particle is assigned to 160-nm (by considering the same mass as MWCNT). If the weight fraction of graphene and CNT are *m*_*G*_ and *m*_*C*_ (*m*_*G*_ + *m*_*C*_  = 1), with the assumed geometries and sizes of graphene particles and CNTs, then the total number of CNTs and graphene particles in a specific volume is 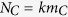 and 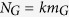 with an arbitrary proportional constant of *k*. Equation (5) can then be evaluated term-by-term. Figure 2a shows the calculated number of CNT-to-CNT, graphene-to-graphene, and CNT-to-graphene contacts. As the concentration of CNTs increases, the number of CNT-to-CNT contacts naturally increases, while the number of graphene-to-graphene contacts decreases. For the same mass case, the number of CNT-to-CNT contacts in the CNT-only assembly (i.e., the 100% CNT content case in Fig. 2a) is larger than the number of graphene-to-graphene contacts in the graphene-only assembly (i.e., the 0% CNT content case in Fig. 2a). This analysis suggests there is a high probability that a CNT will contact another CNT because of the high aspect (length/diameter) ratio, and furthermore that CNTs have an advantage over graphene particles concerning contacts. Thus, the total number of contacts in CNT:graphene hybrids increases as the CNT content increases and reaches a maximum when the packing of CNTs and graphene particles is densest. To obtain the CNT fraction at the maximum, Equation (5) is rewritten into a parabolic equation as a function of CNT content as follows:


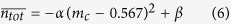


where *α* and *β* are positive constants. In this system, 56.7 wt% is the CNT fraction that can bring about the maximum number of contacts in the modeled CNT:graphene hybrids. At this concentration, the proportion of graphene-to-CNT contact is the largest (75.0%), followed by CNT-to-CNT (19.2%), and graphene-to-graphene (5.8%) contacts. A synergistic behavior of CNT:graphene hybrids can be observed at this CNT content.

Next, the effect of graphene particle size on the number of contacts was investigated. For simplification, the size of the graphene particles was assumed to be 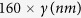. The optimal CNT:graphene mass composition depends on the graphene particle size. Since we assumed the total mass of the CNT:graphene hybrids is fixed, if the mass fraction of graphene particles is 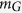, the number of graphene particles can be expressed as 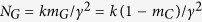 when the radius of the graphene particle changes from 160 nm to 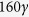 nm. The total number of contacts is then given by:


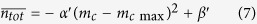


where 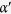 and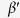 are positive constants and 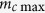 represents the CNT content with the maximum number of contacts. Figure 2b shows the change in this quantity as a function of graphene particle size. As the radius of the graphene particle increases in the hybrid, the CNT content providing the maximum number of contacts also increases. However, this number levels off at a certain graphene size, suggesting that the CNT content does not need to further increase to maintain this maximum number of contacts in a hybrid containing large-sized graphene particles. The number of contacts itself decreases as the graphene particle size increases (Fig. 2c).

The effect of CNT size on the number of contacts can be investigated by changing either its diameter or length. For simplicity, the mass of a CNT is assumed to be independent of its diameter and proportional to its length. By changing the length from 5 to 5*x* μm, the number of CNTs can be expressed as 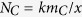, assuming that the total mass of CNTs is fixed. Introducing this into Equation (5) reveals that the number of contacts is not influenced by the CNT length. Next, the effect of changing the CNT diameter is explored. The CNT content for the maximum contacts varies as shown in Fig. 2b. As the diameter of a CNT increases, the CNT content for maximum contacts increases. Additionally, the number of contacts at its maximum increases with increasing CNT diameter.

The number of contacts that graphene and CNT particles can make in their hybrids can thus be calculated using a simplified geometry for the CNT and graphene. The model demonstrates that the condition of maximum contact occurs at a specific concentration of both materials. We can deduce that properties sensitive to such contact, e.g., electrical conductivity (see Eq. (1)), will be maximized at that composition. We prepared randomly-mixed CNT:graphene hybrids using inkjet printing technology to validate this hypothesis.

### Electrical conductivity of CNT:graphene hybrids

There are several requirements for inks to be printed acceptably by an inkjet printer. The inks should have relatively low viscosity (e.g., 1–30 cP) and low volatility (boiling point >100 ^o^C)^45^ Furthermore, the particles in the inks should be smaller than 1/100 times the nozzle diameter (e.g., 200 nm in the printer used in this study) to prevent nozzle clogging. The Methods section provides details of the various graphene and CNT inks that were prepared; each ink viscosity was within the printable range (Supplementary Figure 4). The particle sizes measured using dynamic light scattering (DLS) showed that those inks prepared with less poly(acrylonitrile) (PAN) (used as a dispersion aid for the graphene and CNT particles) had smaller particle sizes. Graphene and CNT inks prepared with graphene:PAN fractions of 1:0.25, 1:0.5, 1:1, and 1:2 and with CNT:PAN fractions of 1:0.25, 1:0.5, 1:1, and 1:2, respectively, had average particle sizes <200 nm (Supplementary Figure 5). CNT and graphene inks containing less PAN had higher zeta potentials and smaller particle sizes (Supplementary Figure 6). Those graphene and CNT inks with graphene:PAN and the CNT:PAN fractions of 1:0.25 were selected for inkjet printing because they satisfied the ink requirements and were expected to have greater electrical conductivity because of the smaller amount of PAN. Thermogravimetric analysis (TGA) determined the weight fractions of graphene and CNT in the graphene and CNT inks with respect to the solvent as 0.394 and 0.178%, respectively (Supplementary Figure 7). Details of the inkjet printer and printing conditions are provided in the Supplementary Information (Supplementary Figure 8).

**Figure 3:**
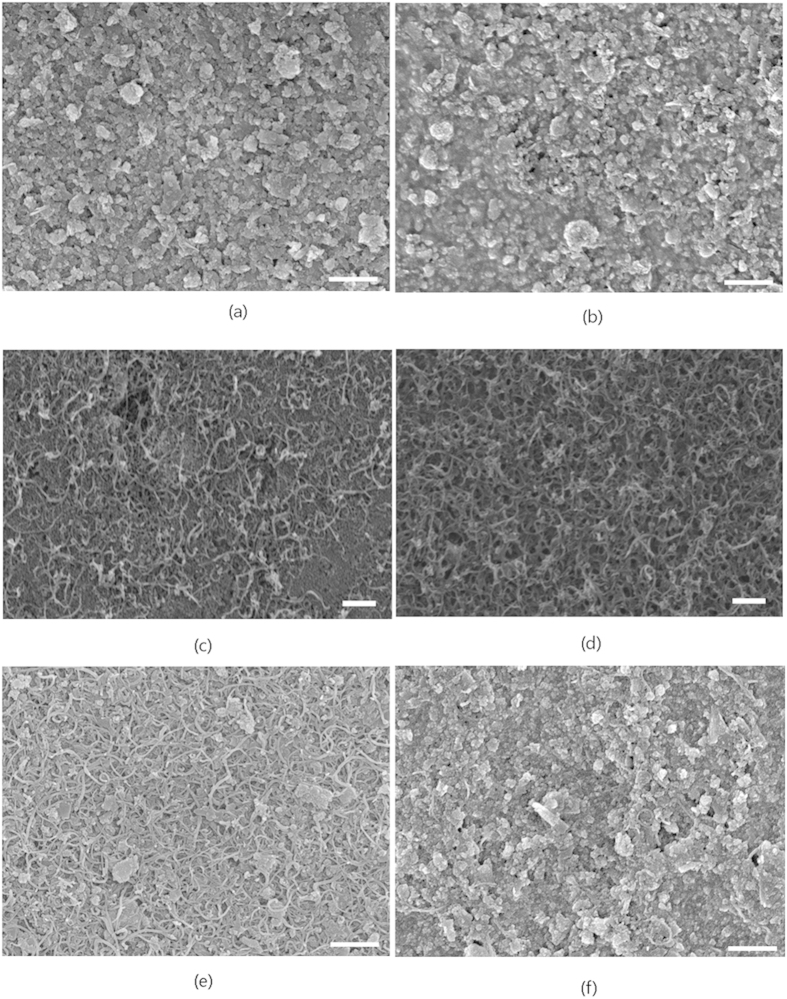
SEM images of graphene particles, CNTs, and their hybrid inks printed on photo paper. The scale bar is 500 nm in all images. (**a,b**): Graphene inks printed 3 and 15 times. The graphene particles were randomly deposited on the paper. The PAN molecules attached to the graphene gave a rough surface to the printed ink. The printed layer was densified as the number of printings increased. (**c,d**): CNT inks printed 3 and 15 times. The CNTs were randomly oriented and distributed on the substrate. (**e,f**): Morphologies of hybrid inks prepared with the CNT fractions of 12 and 81 wt% after printing 15 times. The graphene and CNT particles were randomly distributed and oriented by the inkjet printing process and had multiple interparticle contacts.

The morphologies of the printed inks on photo paper were analyzed using scanning electron microscopy (SEM). The graphene particles were randomly deposited on the paper (Fig. 3a). The PAN molecules attached to the graphene gave a rough surface to the printed ink. Figure 3b shows that the printed layer densified as the number of printings increased. Figure 3c,d show the morphologies of the CNT inks printed 3 and 15 times, respectively. The CNTs were randomly oriented and distributed on the substrate, suggesting that the printed CNTs exhibited isotropic properties. The packing densities of the printed CNT and graphene particles increased with increasing numbers of printings, implying that the conducting path also increased. Figure 3e,f show SEM images of the graphene and CNT hybrid inks that were printed 15 times; the inks contained 12 and 81 wt% of CNTs, respectively. The graphene and CNT particles were randomly distributed and oriented by the inkjet printing process and had multiple interparticle contacts.

**Figure 4:**
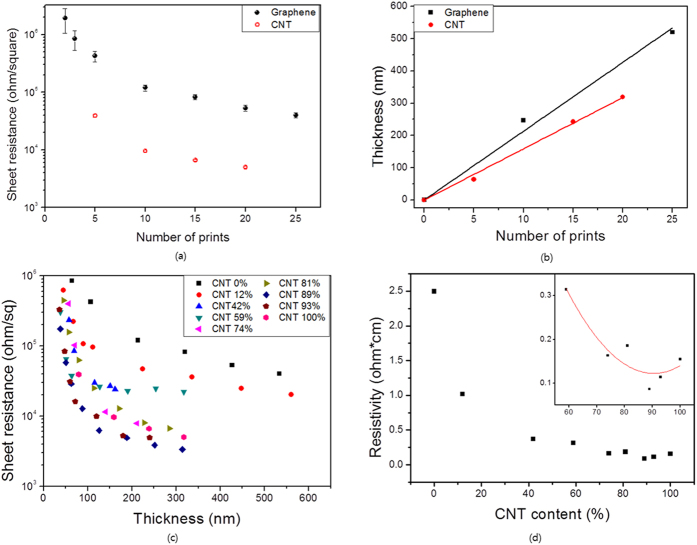
Electrical properties of printed graphene and CNT inks. (**a**) Decreased sheet resistance of printed graphene and CNT inks as the number of printings increased. (**b**) Thickness of printed graphene and CNT inks as a function of the number of printings. (**c**) Sheet resistance of CNT:graphene hybrid inks as a function of the printed thickness and the CNT content. (**d**) Resistivity of the printed hybrid inks as a function of the CNT content, demonstrating a synergistic effect, i.e., the resistivity of the hybrid ink is lower than that of either pure CNT ink or graphene ink.

The electrical conductivity of printed ink was measured using the four-point probe method. Figure 4a shows the sheet resistance of the printed graphene and CNT inks. The printed films prepared with a small number of printings were not electrically conductive because the concentration of conductive particles was insufficient to form conducting paths. The sheet resistance of the printed inks decreased as the number of printings increased. The electrical conductivities of the various inks were compared by measuring the thickness of a printed film by atomic force microscopy (AFM) (Supplementary Figure 9). The thickness of the printed inks linearly increased with increasing number of printings. The thickness of the printed film was 21.3 nm and 15.9 nm per printing for the CNT and graphene inks, respectively (Fig. 4b). Thickly printed inks had a constant conductivity for all thicknesses and thus resembled the bulk material, whereas thinly printed inks showed a percolation threshold, i.e., the conductivity decreased as the thickness decreased. The sheet resistances of the printed graphene and CNT inks at the thickness of 320 nm were 82000 and 5000 Ω sq^–1^, respectively. Although pure graphene has a higher electrical conductivity than pure CNTs^42^,^43^, in this work the CNT inks had higher electrical conductivities than pure graphene because the CNTs formed a better conductive network. The electrical conductivities of the printed CNT and graphene inks were lower than published elsewhere^39^,^46^, this was attributed to the presence of PAN in the printed film. TGA showed that the weight fraction of PAN with respect to the solvent was 0.28 and 0.56 wt% for the graphene and CNT inks, respectively, which are not negligible amounts. PAN improved the dispersion of graphene and CNT particles in solution but it also blocked the conductive paths between particles. Reducing the amount of PAN without disrupting the stability of the inks will improve the electrical properties of the printed inks.

The electrical properties of the CNT:graphene hybrid inks are shown in Fig. 4c–d. All of the hybrid inks printed on the photo paper exhibited decreased sheet resistance as the number of printings increased. The resistivity of the printed ink was calculated by linearly fitting the sheet resistance to 1/thickness. The resistivity of the printed hybrid inks decreased as the CNT content increased, because the increased number of interparticle contacts (see Fig. 2a) expanded the conductive network. The resistivity of the printed hybrid inks exhibited a minimum lower than that of pure CNT ink at a given CNT content. The resistivities of printed graphene and CNT inks were 2.5 and 0.154 Ω cm, respectively, while that of the printed hybrid ink with a CNT content of about 89% was 0.087 Ω cm. This synergistic effect was predicted in the contacts calculation, i.e., that the number of CNT-to-CNT, graphene-to-graphene, and CNT-to-graphene interparticle contacts would be at a maximum at a specific CNT content. Figure 2b shows the effect of CNT diameter and graphene radius on CNT content at the maximum number of contacts. The calculations indicated that the CNT:graphene hybrid made from CNTs and graphene particles with certain sizes would have a maximum number of contacts at a CNT content of 89% (the detailed derivations are provided on section 8 in the supplementary information). The diameter of CNT and radius of graphene when the maximum number of contacts at a CNT content of 89% occurs is shown in the Fig. 2d. The approach used in this study is thus suitable for investigating the properties of CNT:graphene hybrids, in particular the electrical conductivity.

## Discussion

The number of CNT-to-CNT, graphene-to-graphene, and graphene-to-CNT interparticle contacts was calculated using an orientation density function of graphene and CNT in a hybrid assembly. CNTs and graphene particles were assumed to be straight cylinders and disks, respectively. The probability of each type of contact was then calculated, from which the number of contacts was obtained. The total number of contacts followed a parabolic equation as a function of CNT concentration, suggesting the existence of a maximum at a specific composition ratio. This indicates a synergistic effect of the properties of the CNT:graphene hybrids, in particular those properties that are contact-dependent. This synergistic effect was experimentally investigated by using inkjet printing to prepare CNT:graphene hybrids. Hybrid inks, prepared at a certain composition ratio, had higher electrical conductivities than those of pure CNT or graphene, i.e., a synergistic effect. These findings support the use of our statistical approach to investigate the properties of particulate hybrid materials involving contacts of the constituent elements.

Our model was aimed to estimate the electrical conductivity of CNT:graphene hybrids. However, we have found out that the percolation threshold of CNT composites can be estimated using the equations derived in our work. In previous research, the critical volume fraction ensuring the percolation was estimated by calculating the probability of one cylinder intersecting at least two other cylinders. In a work by Munson-McGee, the critical volume fraction was chosen when the probability that one cylinder intersecting at least two other cylinder is 0.5^47^. The probability that one cylinder contact another cylinder was obtained by dividing the excluded volume by the total volume where the cylinders can be located as in our work. We assume that each CNT in the conducting paths must have at least 2 contacts with other CNTs. We set the critical concentration of percolation when the average number of contacts becomes 2. The result shows that the critical volume fraction of CNTs is the inverse of the aspect ratio of CNTs (supplementary Figure 11). The derivation and more information about this are provided in the supplementary information.

The size distribution of graphene particles and CNTs can be considered in the calculation of the number of contacts through size distribution function. The number of CNT-to-CNT, graphene-to-graphene, and graphene-to-CNT contacts can be derived as follows (detailed derivations are provided in the supplementary information).


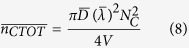



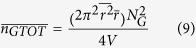



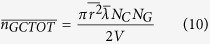


Note that the overlines in the equation represent the mean value of a quantity, e.g., the mean length and diameter of CNTs and the mean radius of graphene flakes. As shown in the Equation (8, 9, 10), the number of contacts in the CNT:graphene hybrids is related to the mean length and diameter of CNT and the mean radius and mean square of the radius of graphene.

## Methods

### Materials and Inks

Graphene particles (XG Sciences, Grade C750), multi-walled carbon nanotubes (MWCNT, Hanwha Nanotech, CM-95), poly(acrylonitrile) (PAN, Polysciences, homopolymer, MW 200000), and N,N-dimethylformamide (DMF, Daejung Chemical, EP grade, 99.5%) were used to prepare CNT and graphene hybrid inks using the following process. First, PAN solutions were prepared at 0.25, 0.5, 1, and 2 wt% concentrations in DMF (70 g). The solutions were stirred on a hot plate at 90 °*C*. Graphene (0.7 g) was then added to each PAN solution. The graphene solutions were stirred and then ultrasonicated. Then, the solutions were centrifuged at 5000 rpm for 15 min and the supernatant was vacuum-filtered through filter paper having a pore size of 1.2 μm (GF3-grade glass microfiber filter, Whatman). CNT and graphene inks were prepared in the same manner. The separate graphene and CNT inks were then mixed together in various proportions to make different CNT:graphene hybrid inks.

### Characterization and Inkjet Printing

The viscosity of each solution was measured using a rheometer (AR–G2, TA Instruments) at shear rates of 4.642, 10, 21.55, 46.42, 100, 215.5, and 464.2 s^−1^ at 25 °*C* to adjust the printing parameters. The average size of the particles in an ink was measured using a dynamic light scattering spectrophotometer (DLS–7000, Otsuka Electronics). The zeta potential was also measured using an electrophoretic light scattering spectrophotometer (ELS–8000, Otsuka Electronics) to evaluate the degree of dispersion of the particles. TGA was carried out to calculate the amount of graphene, CNT, and PAN in the inks.

An inkjet printer (Dimatix DMP–2800, Fujifilm) was used to print the prepared inks on a substrate. The droplet formation of the inks was controlled by changing the cartridge settings, including the jetting voltage, waveform, meniscus control, and temperature. Photo paper was selected as the substrate. The inks were printed in a square pattern 7 × 7 mm^2^ with a drop spacing of 25 μm, varying the number of printings from 1–25. The thicknesses and morphologies of the printed inks were investigated using AFM and SEM, respectively. The electrical properties of the printed inks were measured using the four-point probe method.

## Additional Information

**How to cite this article**: Shim, W. *et al.* Optimally conductive networks in randomly dispersed CNT:graphene hybrids. *Sci. Rep.* **5**, 16568; doi: 10.1038/srep16568 (2015).

## Supplementary Material

Supplementary Information
