# Supplementary material for: Optimally conductive networks in randomly dispersed CNT:graphene hybrids
Source: Sci Rep. 2015 Nov 13;5:16568. doi: 10.1038/srep16568 (PMC4643282; doi:10.1038/srep16568)
Supplement: Supplementary Information [file srep16568-s1.doc]

Supporting Information to

**Optimally conductive networks in randomly dispersed CNT:graphene hybrids**

*Wonbo Shim, Youbin Kwon, Seung-Yeol Jeon, and Woong-Ryeol Yu[[1]](#footnote-2),**

Department of Materials Science and Engineering and Research Institute of Advanced Materials (RIAM), Seoul National University, Seoul, KOREA

***1. Estimating the number of CNT-to-CNT contacts***

The number of contacts between CNTs can be calculated using the method proposed by Komori and Makishima^1^ for calculating the number of fiber-to-fiber contacts in fiber assemblies. The fibers are assumed to have straight cylindrical geometries. Their approach was used here to calculate the number of CNT-to-CNT contacts by simply replacing fibers with CNTs in the model. The numbers of CNT-to-graphene and graphene-to-graphene contacts were also calculated by modifying the model. The original theory is briefly summarized in this Supporting Information; further details can be found in^1^.

The orientation of a fiber can be expressed by two angles, i.e., a pair (
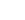
) where
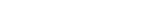
 represents an angle between the *z-*axis and the axis of the fiber and between the *x*-axis and the normal projection of the fiber axis onto the *xy* plane (see Figure 1 in the main manuscript). The probability of finding the orientation of a fiber in the infinitesimal range of angles
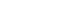
 and
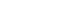
 is
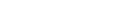
, where
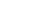
 is the orientation density function. The following condition must be satisfied for the probability density function:

(S1)

For the random orientation case, Ω becomes a constant (1/2π). We consider two CNTs, (*A*) and (*B*) having CNT orientations of and , respectively, in arbitrary positions in a volume *V*. CNT *B* will contact CNT *A* when the center of mass of CNT *B* enters into the region shown in Supplementary figure 1. If the diameter and length of the CNT are
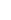
 and
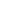
, respectively, then the volume of this region (
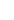
) becomes:

(S2)

where
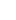
 is the angle between the two CNTs.


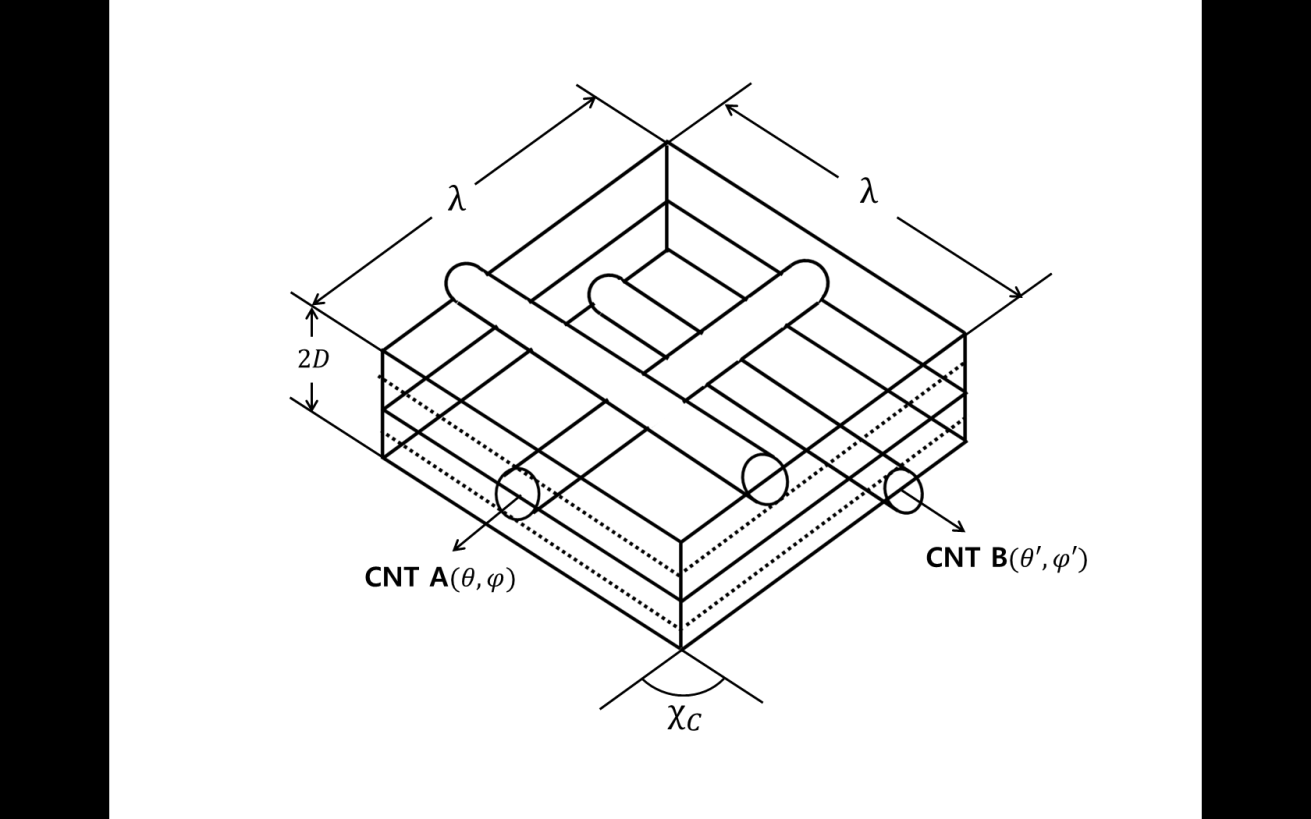


Supplementary figure 1. The parallelepiped formed by a CNT *B*
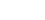
 moving around a fixed CNT *A*
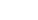
 while maintaining contact^1^.

The probability that CNT *B* contacts CNT *A* is then given by:

(S3)

If there are *N_c_* CNTs in the volume *V*, the average number of CNTs that come into contact with CNT *A* is calculated as:

(S4)

For large *N_c_* (*N_c_* >> 1):

(S5)

where:

(S6)

The average number of contacts
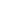
 on an arbitrary CNT having a random orientation is:

$\overline{n_{C}}=n\left(0,0\right)=\frac{2D\lambda^{2}N_{C}}{V}J\left(0,0\right)=\frac{\pi D\lambda^{2}N_{C}}{2V}$ (S7)

The total number of the contacts in a CNT assembly of volume *V* containing *N_c_* CNTs in a random orientation is obtained by multiplying
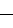
 by
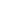
. The factor 1/2 avoids double- counting of the contacts. Finally, the following equation can be obtained to calculate the number of CNT-to-CNT contacts:

$\overline{n_{Ctot}}=\frac{\pi D\lambda^{2}N^{2}_{C}}{4V}$ (S8)

Equation (S8) can be directly used to calculate the number of CNT-to-CNT contacts, assuming CNTs are straight cylinders with a constant length and diameter.

***2. Estimating the number of graphene-to-graphene contacts***

To estimate the number of graphene-to-graphene contacts, we considered graphene to be a disk with a radius of
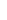
 and negligible thickness. We oriented a graphene particle *A* with its normal vector having an orientation of
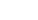
 placed in an arbitrary position in volume *V*. Similar to the CNT-to-CNT contact case, another graphene particle *B* with orientation (
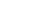
) was considered to contact graphene *A* when the center of mass of *B* enters into the region shown in Supplementary figure 2. The volume
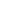
 of this region is given by:

(S9)

where
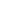
 is the angle between the normal vectors of graphene particles *A* and *B*.

The probability that graphene particles *A* and *B* contact is:

(S10)

If there are *N_G_* graphenes in a volume *V*, the average number of graphenes that come into contact with the graphene particle *A* is:

(S11)

where
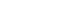
 is the orientation density function. For large *N_G_* (*N_G_* >> 1), the method used for the derivation of Equations S5–S7 gives the total number of graphene-to-graphene contacts in the assembly (
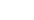
 ) as:

$\overline{n_{Gtot}}=\frac{\left(2\pi^{2}r^{3}\right)N^{2}_{G}}{4V}$ (S12)


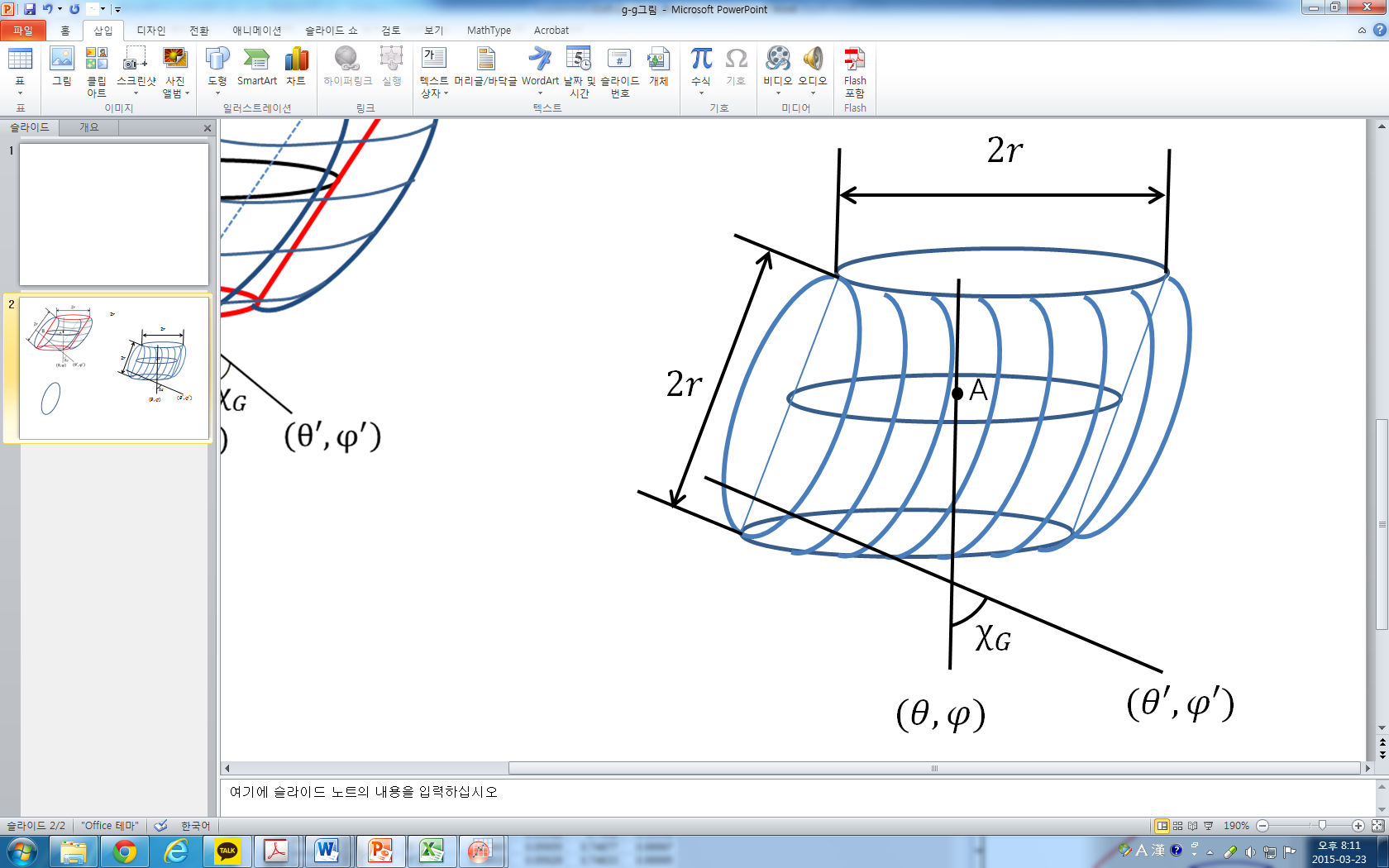


Supplementary figure 2. A schematic drawing for calculating the contact probability of graphene particles *A* and *B*. Mobile graphene particle *B* will contact fixed particle *A* when the center of mass of *B* enters the region surrounded by the blue and red lines.

***3. Estimating the number of graphene-to-CNT contacts***

The number of graphene-to-CNT contacts was calculated in a similar way to that used for the CNT-to-CNT and graphene-to-graphene contacts. Consider that arbitrary graphene particle *A* having an orientation of
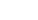
 is placed in a volume *V*. A CNT with an orientation of
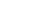
 will contact the graphene particle *A* when the center of mass of the CNT enters the region surrounded by the dotted lines shown in Supplementary figure 3. The volume of this region
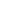
 can be calculated by:

(S13)

where
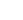
 is the angle between the CNT axis and the normal vector of the graphene particle.


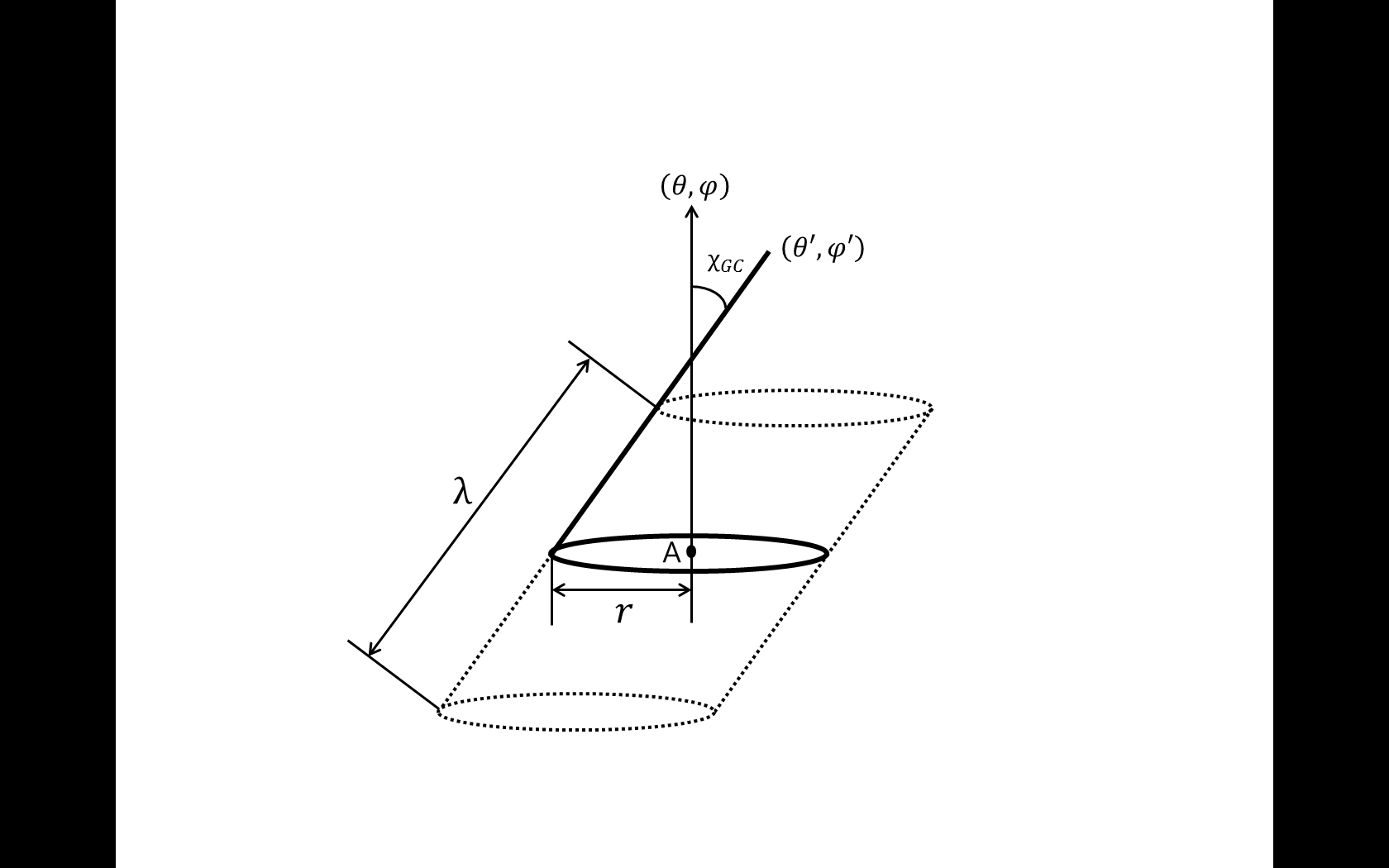


Supplementary figure 3. A schematic diagram for explaining the contact condition of a CNT with graphene particle *A*. The CNT will contact *A* when the center of mass of the CNT enters into the region surrounded by the dotted lines.

The probability
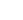
 that a CNT will contact *A* is:

(S14)

The average number of contacts between CNTs and *A* when a total of *N_C_* CNTs exist in the graphene:CNT assembly in a volume V is:

(S15)

Using the same approach as that used in the derivation of Equations (S5)–(S7), the total number of graphene-to-CNT contacts (
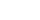
) in the assembly of volume *V* with *N_C_* CNTs and *N_G_* graphenes is:

$\overline{n_{GCtot}}=\frac{\pi r^{2}\lambda N_{C}N_{G}}{2V}$ (S16)

***4. Viscosities, particle sizes, and zeta potentials of CNT:graphene ink***

(a)

(b)

Supplementary figure 4. Viscosities of (a) CNT ink and (b) graphene ink.

(a)

(b)

Supplementary figure 5. Particle sizes of PAN-wrapped CNTs and graphene particles measured using DLS as a function of PAN content.

(a)

(b)

Supplementary figure 6. Zeta potentials of PAN-wrapped CNTs and graphene particles measured using ELS as a function of PAN content.

***5. Weight fraction of CNT:graphene in the ink***

The weights of CNT and graphene inks were measured before and after evaporation of the solvent to measure the weight fraction of CNT:graphene particles in the inks. Poly(acrylonitrile) (PAN) was used as a modifier to improve the dispersion of CNTs and graphene particles. First, the weight fractions of PAN + graphene and PAN + CNTs were determined to be 0.547 and 0.292 wt% for the pure graphene and CNT inks, respectively. The weight fractions of graphene/(graphene + PAN) and CNT/(CNT + PAN) were determined using thermogravimetric analysis (TGA) (Supplementary figure 7). The decrement of each material at 800 ^o^C was used to calculate the weight fraction as follows. If the weight fractions of graphene or CNT and PAN are *x* and *y* (*x* + *y* = 1), respectively, then the following equation can be derived:

$\begin{matrix} \left(wt\%\text{ decrement of graphene/CNT}\right)x+\left(wt\%\text{ decrement of PAN}\right)y \\ =\left(wt\%\text{ decrement of dried inks}\right) \end{matrix}$ (S17)

The weight fractions of graphene and CNT with respect to graphene + PAN and CNT + PAN were 0.72 and 0.44, respectively. This indicates that significant amounts of PAN were present, and accounts for the low electrical conductivities of the printed inks. The weight percentages of graphene and CNTs in the graphene and CNT inks with respect to the weight of the solvent were 0.394 and 0.178 %, respectively.

(a)

(b)

Supplementary figure 7. TGA curves of (a) CNT and (b) graphene inks.

***6. Printing of CNT and graphene inks on photopaper***


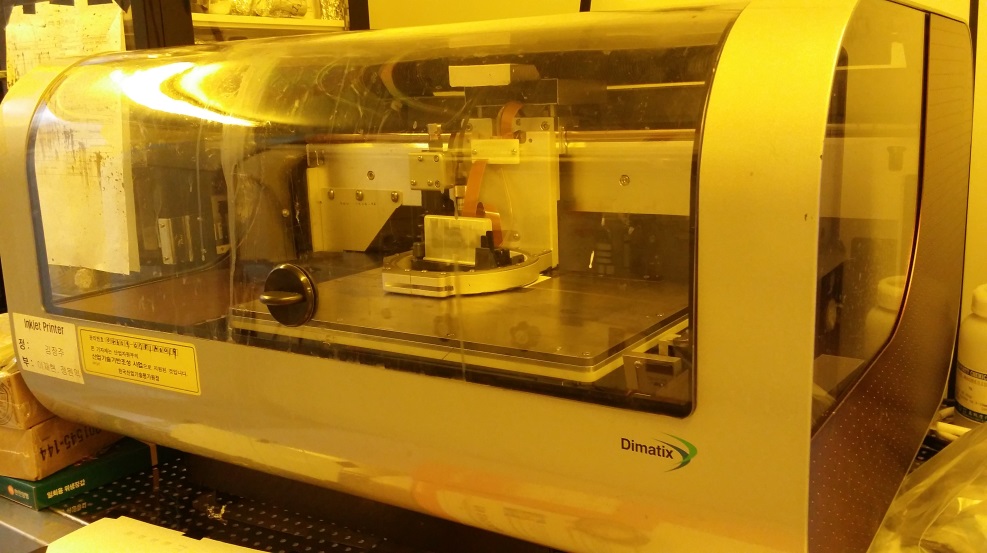


(a)


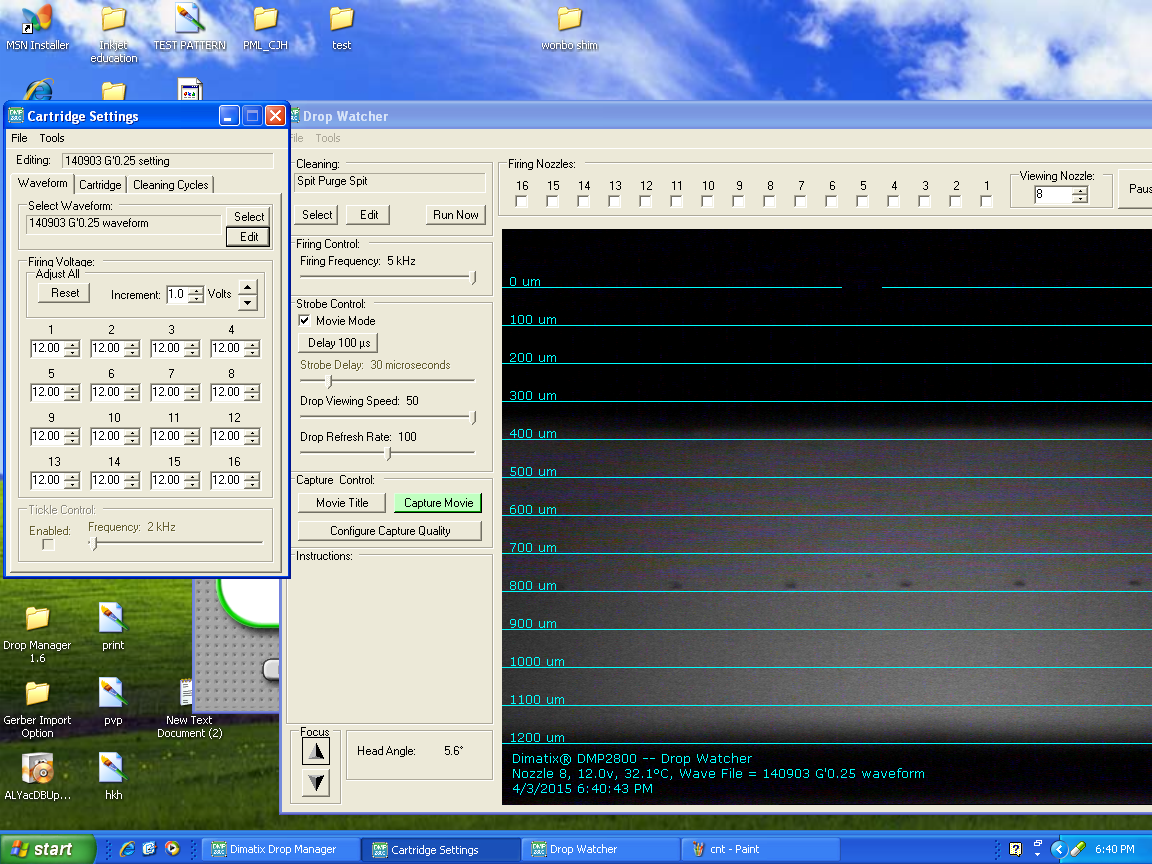


(b)


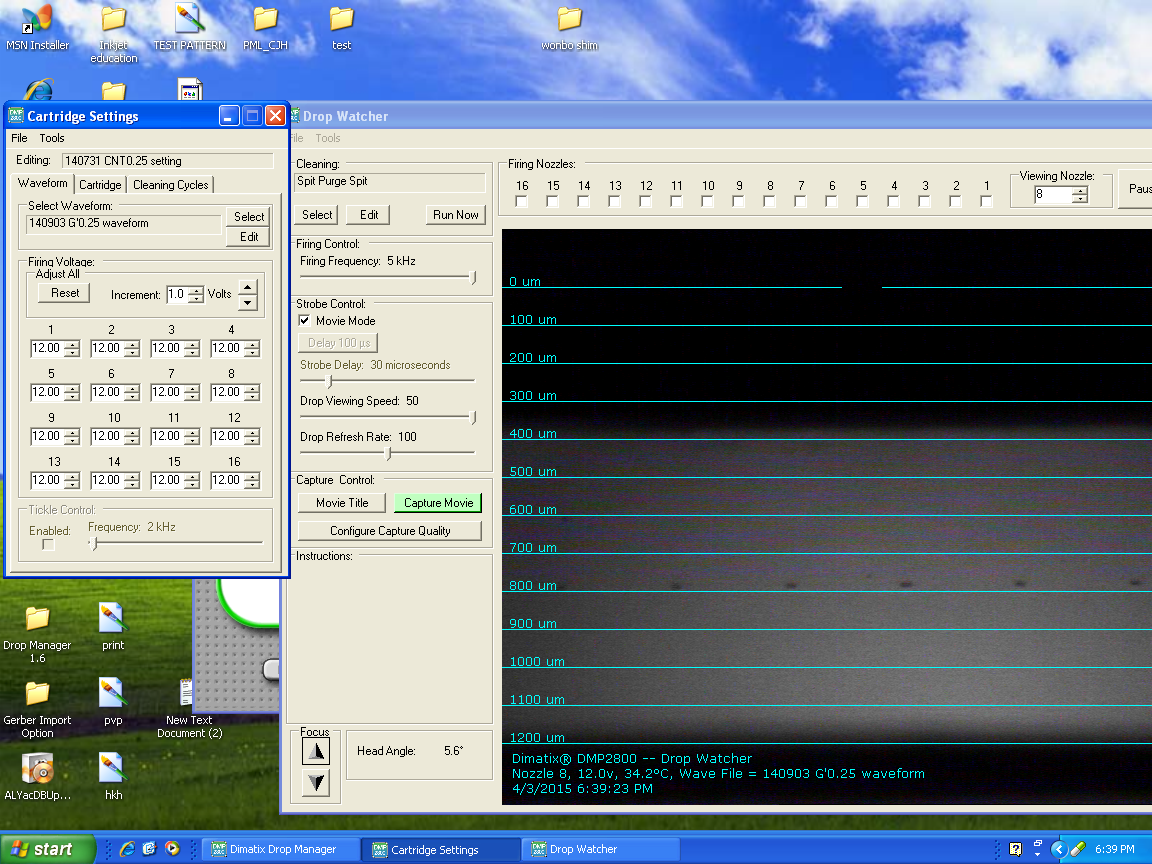


(c)


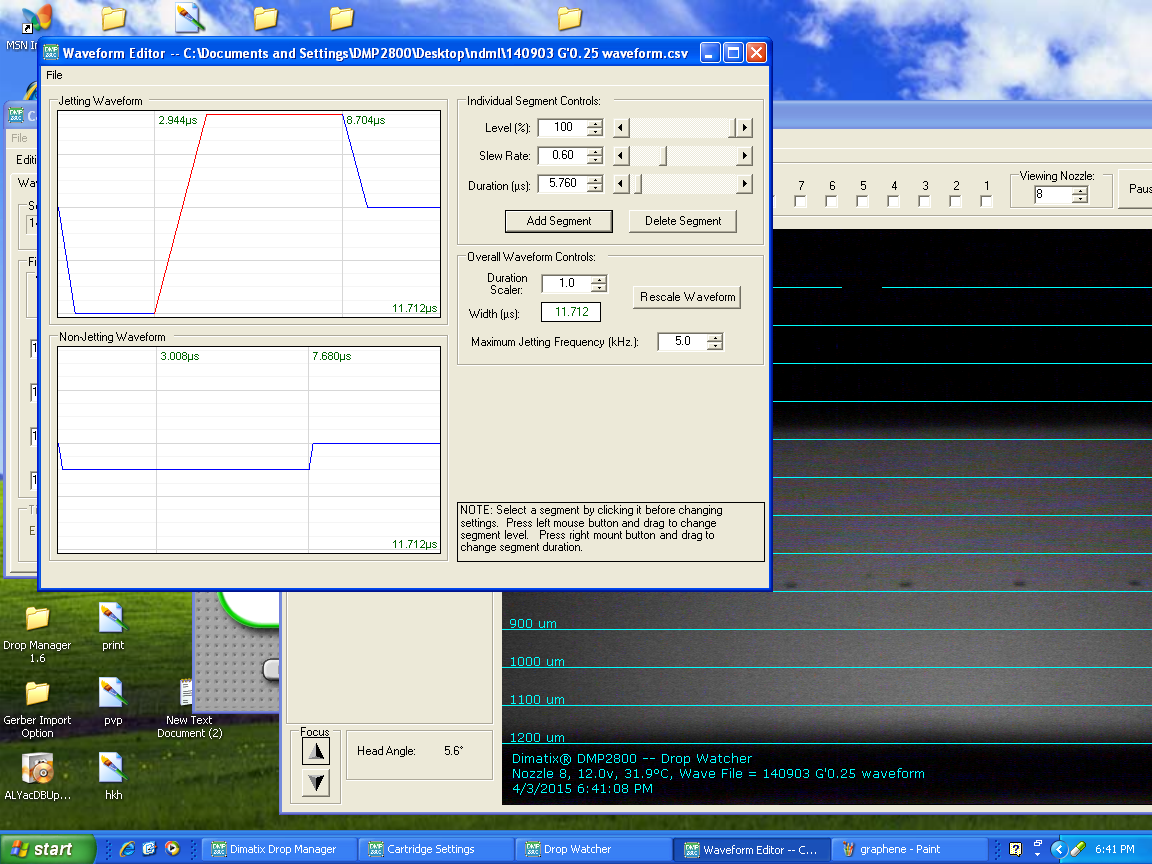


(d)


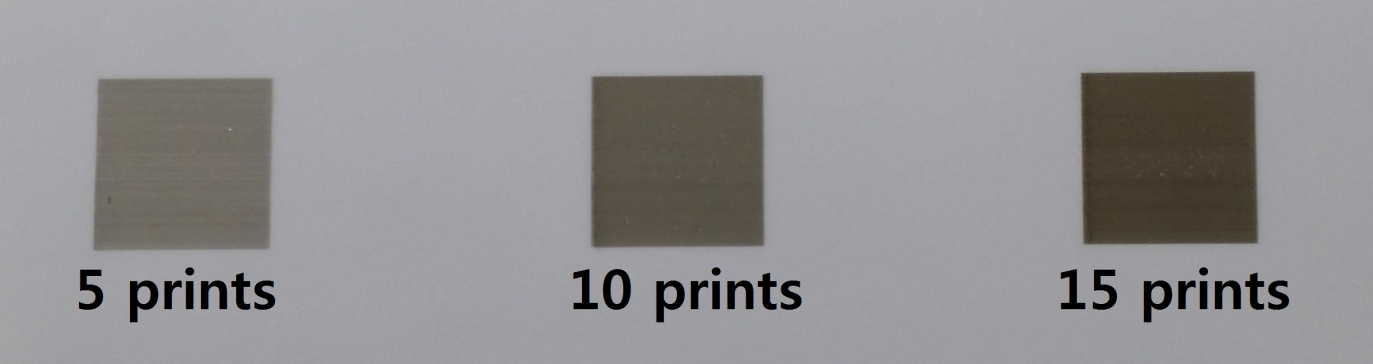


(e)

Supplementary figure 8. (a) The inkjet printer used in this study (Dimatix DMP–2800, Fujifilm). Jetting voltage conditions of the (b) graphene ink and the (c) CNT ink, and (d) waveform setting conditions. (e) The inks were printed on the photopaper in a square pattern of 7 mm × 7 mm^2^.

***7. Thickness characterization of the printed inks***


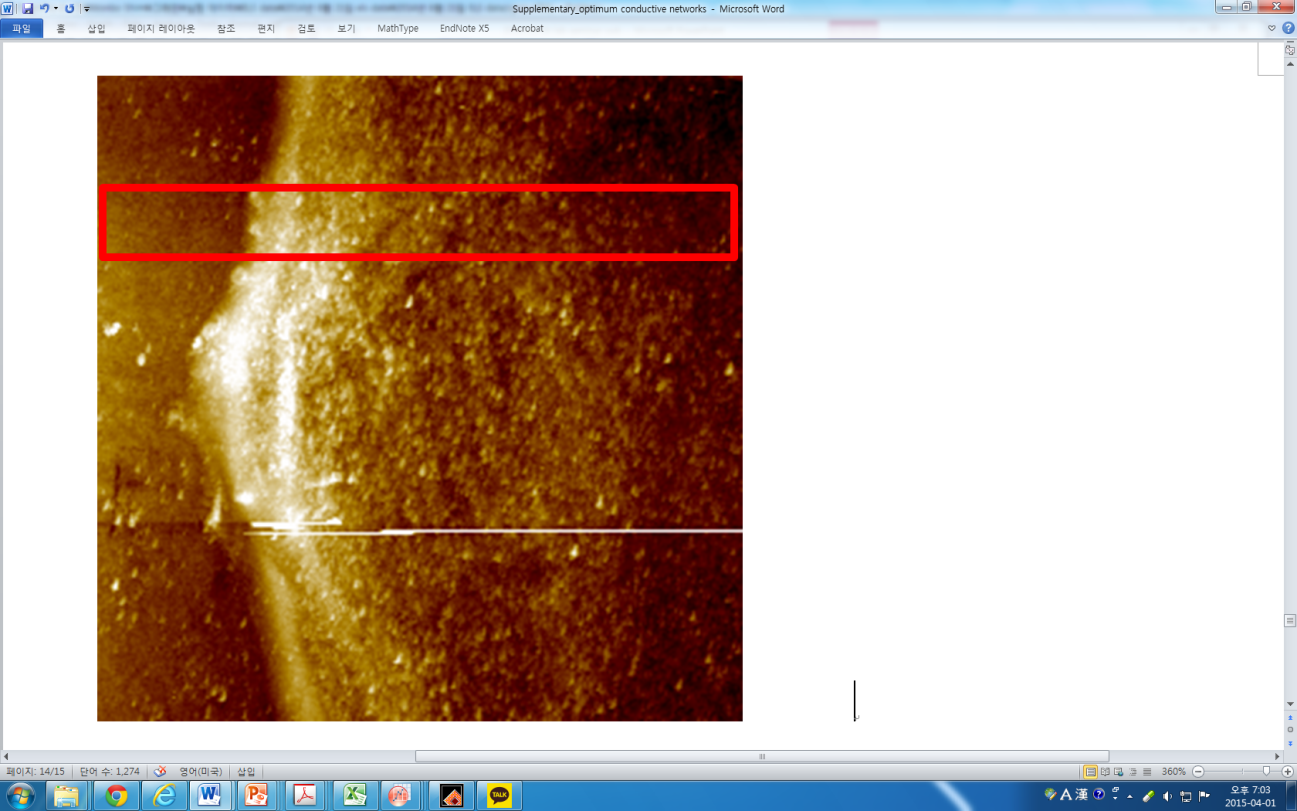


(a)


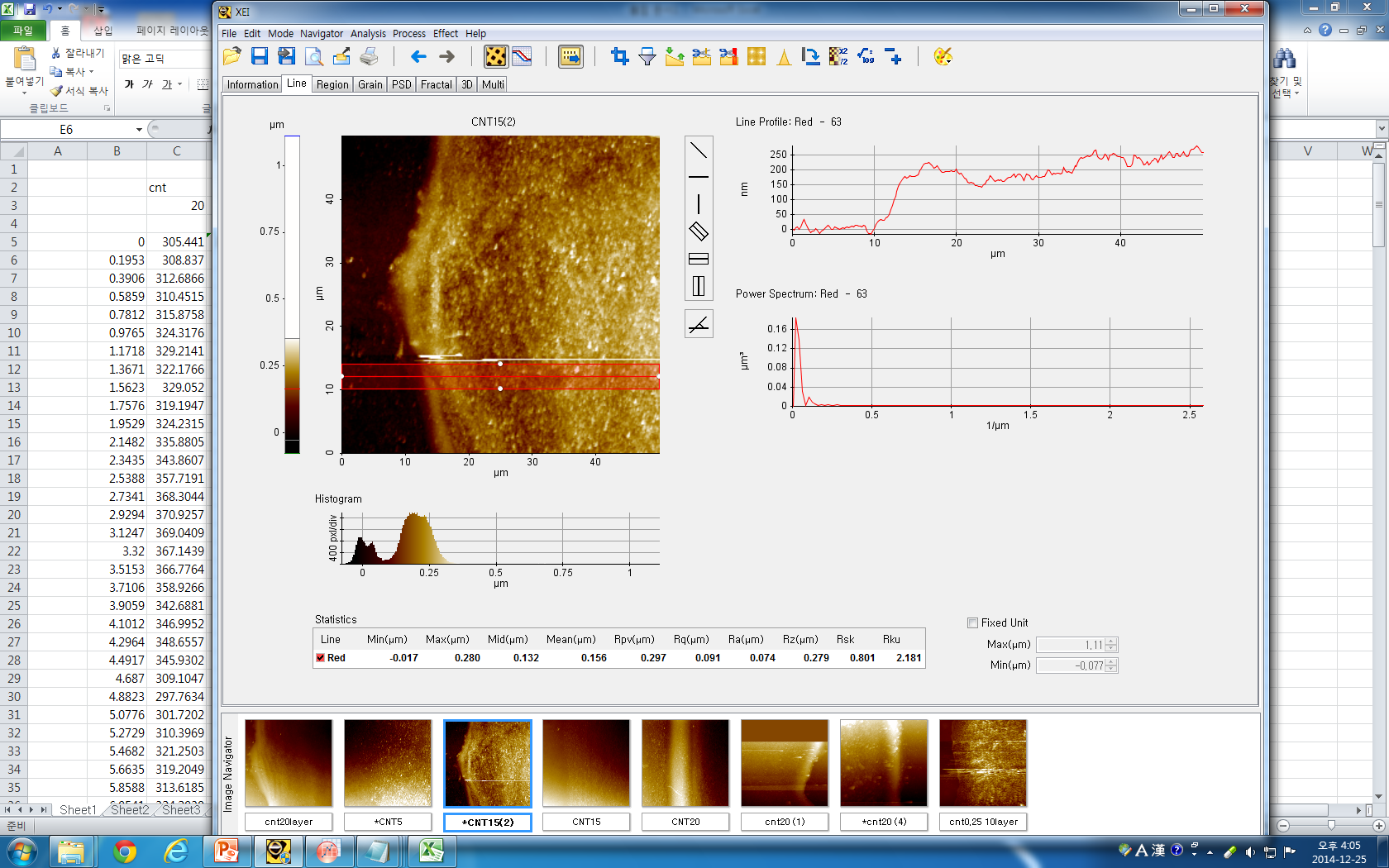


(b)

Supplementary figure 9. (a) An AFM image of a printed CNT ink and (b) heights of the region surrounded by the red rectangle shown in (a).

***8. Derivations for calculating the size of graphene and CNT when the maximum number of contacts occurs at certain compositions.***

We apply
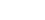
 μm,
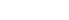
 μm,
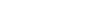
 and
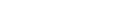
 into Equation (5), resulting in Equation (S18).

$\overline{n_{tot}}=\frac{\pi k^{2}}{4V}(25Dm_{C}^{2}+\frac{0.0257}{\gamma}(1-m_{C})^{2}+0.256m{}_{C}(1-m_{C}))$ (S18)

The CNT weight fraction at the maximum number of contacts (
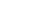
) can be calculated when Equation (S18) is simplified into Equation (7) form and
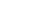
 is given by:

$m_{C\max}=\frac{0.0514/\gamma-0.256}{50D-0.512+0.0514/\gamma}$ (S19)

In case of experimental results in our work, the maximum number of contacts occurred when the CNT content was 89%. When 89% is applied to
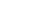
, the relation between
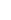
 and
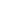
 can be obtained:

$D=\frac{1.28\times10^{-4}}{\gamma}+4.5\times10^{-3}\mu m$ (S20)

Since
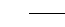
, Equation (S20) can be expressed in terms of the diameter of CNT and the radius of graphene as follows.

$D=\frac{20.48}{r}+4.5nm$ (S21)

This relation is plotted in supplementary figure 10.

Supplementary figure 10. Relation between the diameter of CNT and radius of graphene when the maximum number of contacts occurs at CNT content of 89%

***9. Calculation of percolation threshold for CNT assembly***

Our model was aimed to estimate the electrical conductivity of CNT:graphene hybrids. We have found out that the percolation threshold of CNT composites can be estimated using the equations derived in our work. In previous research, the critical volume fraction ensuring the percolation was estimated by calculating the probability of one cylinder intersecting at least two other cylinders. In a work by Munson-McGee, the critical volume fraction was chosen when the probability that one cylinder intersecting at least two other cylinder is 0.5.^2^ The probability that one cylinder contact another cylinder is obtained by dividing the excluded volume by the total volume where the cylinders can be located as in our work. Following Munson-McGee’s work, the critical volume fraction can be obtained using our approach (the number of contacts) as follows.

We assume that each CNT in the conducting paths must have at least 2 contacts with other CNTs. We set the critical concentration of percolation when the average number of contacts becomes 2. The average number of contacts on an arbitrary CNT can be expressed by,

$\overline{n}=\frac{\pi D\lambda^{2}N}{2V}\left(=2\right)$ (S22)

The volume fraction of CNT (
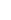
) can be calculated by dividing the total volume of CNTs by the total volume where CNT can be located.

(S23)

The critical volume fraction (
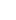
) of the CNTs when the average number of contacts on CNT becomes 2 can be calculated by relating Equations (S22) and (S23), resulting in,

(S24)

As shown in Equation (S24), the critical volume fraction of CNT (
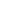
 is the inverse of the aspect ratio. The relation between the aspect ratio of CNT and the percolation threshold is shown in the supplementary figure 11. The calculated percolation threshold here has almost the same value with the percolation threshold calculated in other works.^3^

Supplementary figure 11. The relation between aspect ratio of CNT and percolation threshold.

***10. Estimating the number of contacts for graphene:CNT hybrids composed of graphene and CNT with different sizes.***

In the previous sections 1 through 3, we derived the number of CNT-to-CNT, CNT-to-graphene, and graphene–to-graphene contacts for same sized CNTs and graphene. Here, we extend our approach to cover CNTs and graphene with size distribution. If we consider the size distribution function of CNT length as
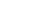
 as in supplementary figure 12, the number of CNT-to-CNT contacts for mixture of CNTs with different sizes can be calculated as follows.


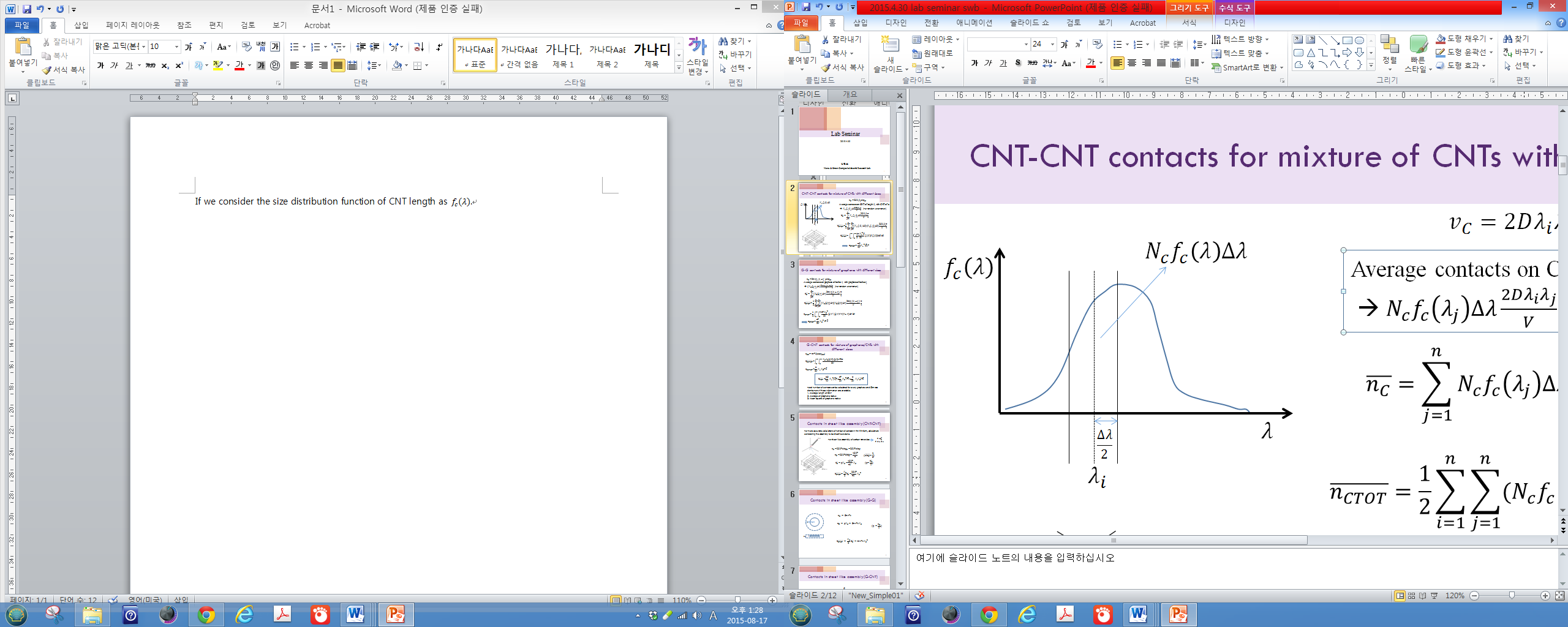


Supplementary figure 12. An example of a graph showing size distribution of CNTs

If we consider the length of two CNTs in supplementary figure 1 as
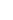
 and
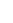
, the volume of the region in this figure becomes

$v_{C}=2D\lambda_{i}\lambda_{j}\sin\chi_{C}$ (S25)

By using the same method from equation (S3)-(S7), the average number of contacts on CNT of length
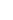
 with CNT of length
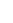
 for random orientation is calculated by:

$N_{C}f_{C}(\lambda_{j})\Delta\lambda\frac{2D\lambda_{i}\lambda_{j}}{V}\frac{\pi}{4}$ (S26)

The average number of contacts on CNT of length
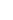
 can be calculated by considering the length of all the other CNTs and is given by:

$\overline{n_{C}}=\sum_{j=1}^{n} N_{C}f_{C}(\lambda_{j})\Delta\lambda\frac{2D\lambda_{i}\lambda_{j}}{V}\frac{\pi}{4}$ (S27)

The total number of contacts can be calculated by multiplying half the number of CNTs of all length and is given by:

$\overline{n_{Ctot}}=\frac{1}{2}\sum_{i=1}^{n} \sum_{j=1}^{n} (N_{C}f_{C}(\lambda_{i})\Delta\lambda)(N_{C}f_{C}(\lambda_{j})\Delta\lambda)\frac{2D\lambda_{i}\lambda_{j}}{V}\frac{\pi}{4}$ (S28)

Equation (S28) can be changed into the following integral form:

$\overline{n_{Ctot}}=\int_{0}^{\infty} \int_{0}^{\infty} \frac{1}{2}\frac{\pi N_{C}^{2}D}{2V}f_{C}(\lambda')\lambda'f_{C}(\lambda)\lambda d\lambda'd\lambda$ (S29)

By integrating Equation (S29), the total number of CNT-to-CNT contacts for CNTs with size distribution is calculated:

$\overline{n_{Ctot}}=\frac{\pi D}{4V}N_{C}^{2}(\overline{\lambda})^{2}$ (S30)

where $\overline{\lambda}$ is the mean length of CNTs. Comparing Equation (S30) with (S8), it is clear that only the mean length of CNTs is necessary for estimating the number of CNT-to-CNT contacts for CNT assembly with size distribution. If we also consider the diameter of CNTs with different sizes,
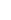
 will be replaced with
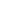
 ( the mean diameter of CNTs) in Equation (S30). Therefore, the total number of CNT-to-CNT contacts is proportional to the average length, average diameter and total number of CNTs.

In case of graphene-to-graphene contacts, we consider the size distribution of graphene radius as
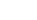
. In supplementary figure 2, if we consider the radius of two graphene particles in contact as
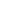
 and
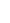
, the volume of the region shown in that figure becomes:

$v_{G}=2\pi r_{i}r_{j}(r_{i}+r_{j})\sin\chi_{G}$ (S31)

By using the same method we have shown above, the average number of contacts on graphene of radius
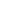
 with graphene of radius
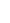
 becomes:

$(N_{G}f_{G}(r_{j})\Delta r)\frac{2\pi r_{i}r_{j}(r_{i}+r_{j})}{V}\frac{\pi}{4}$ (S32)

The average number of contacts on graphene of radius
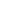
 becomes:

$\overline{n_{G}}=\sum_{j=1}^{n} (N_{G}f_{G}(r_{j})\Delta r)\frac{2\pi r_{i}r_{j}(r_{i}+r_{j})}{V}\frac{\pi}{4}$ (S33)

The total number of graphene to graphene contacts in mixture of graphene particles with different sizes becomes:

$\overline{n_{Gtot}}=\frac{1}{2}\sum_{i=1}^{n} \sum_{j=1}^{n} (N_{G}f_{G}(r_{j})\Delta r)(N_{G}f_{G}(r_{i})\Delta r)\frac{2\pi r_{i}r_{j}(r_{i}+r_{j})}{V}\frac{\pi}{4}$ (S34)

$\overline{n_{Gtot}}=\frac{1}{2}\int_{0}^{\infty} \int_{0}^{\infty} \frac{\pi^{2}N_{G}^{2}}{2V}f_{G}(r')f_{G}(r)r'r(r'+r)dr'dr$ (S35)

By calculating equation (S35), the total number of graphene-to-graphene contacts is obtained and is given by:

$\overline{n_{Gtot}}=\frac{\pi^{2}N_{G}^{2}\overline{r^{2}}\overline{r}}{2V}$ (S36)

Equation (S36) shows that the total number of graphene-to-graphene contacts for different graphene sizes is proportional to the mean radius of graphene, mean square of graphene radius, and the total number of graphene particles.

In case of graphene-to-CNT contacts, if we consider mixture of graphene particles and CNTs with different sizes, the volume of the region in the supplementary figure 3 becomes:

$v_{GC}=\pi r_{i}^{2}\lambda_{j}\left|\cos\chi_{GC}\right|$ (S37)

By using the same method we used above, the total number of graphene-to-CNT contacts is calculated as

$\overline{n_{GCtot}}=\int_{0}^{\infty} \int_{0}^{\infty} \frac{N_{C}N_{G}f_{C}(\lambda)f_{G}(r)r^{2}\lambda\pi}{2V}drd\lambda$ (S38)

Finally, Equation (S38) gives:

$\overline{n_{GCtot}}=\frac{\pi}{2V}N_{C}N_{G}\overline{r^{2}}\overline{\lambda}$ (S39)

Equation (S39) shows that total number of graphene-to-CNT contacts in mixture of graphene and CNT with different sizes is proportional to the mean square of graphene radius, the mean length of CNTs, the number of CNTs and graphene particles.

**References**

1 Komori, T. & Makishima, K. Numbers of Fiber-to-Fiber Contacts in General Fiber Assemblies. *Text. Res. J.* **47**, 13-17 (1977).

2 Munson-McGee, S. H. Estimation of the critical concentration in an anisotropic percolatin network. *Phys. Rev. B* **43**, 3331-3336 (1991).

3 Hu, N. *et al.* Effect of fabrication process on electrical properties of polymer/multi-wall carbon nanotube nanocomposites. *Compos. Part A-Appl. S.* **39**, 893-903 (2008).

1. Corresponding author. Email: woongryu@snu.ac.kr [↑](#footnote-ref-2)
